# Bacterial Community Succession in Pine-Wood Decomposition

**DOI:** 10.3389/fmicb.2016.00231

**Published:** 2016-03-01

**Authors:** Anna M. Kielak, Tanja R. Scheublin, Lucas W. Mendes, Johannes A. van Veen, Eiko E. Kuramae

**Affiliations:** Department of Microbial Ecology, Netherlands Institute of EcologyWageningen, Netherlands

**Keywords:** microbial ecology, 16S rRNA, ITS region, C/N ratio, wood decomposition, bacterial community assembly, fungal community assembly

## Abstract

Though bacteria and fungi are common inhabitants of decaying wood, little is known about the relationship between bacterial and fungal community dynamics during natural wood decay. Based on previous studies involving inoculated wood blocks, strong fungal selection on bacteria abundance and community composition was expected to occur during natural wood decay. Here, we focused on bacterial and fungal community compositions in pine wood samples collected from dead trees in different stages of decomposition. We showed that bacterial communities undergo less drastic changes than fungal communities during wood decay. Furthermore, we found that bacterial community assembly was a stochastic process at initial stage of wood decay and became more deterministic in later stages, likely due to environmental factors. Moreover, composition of bacterial communities did not respond to the changes in the major fungal species present in the wood but rather to the stage of decay reflected by the wood density. We concluded that the shifts in the bacterial communities were a result of the changes in wood properties during decomposition and largely independent of the composition of the wood-decaying fungal communities.

## Introduction

Degradation of plant remains is important in carbon and nutrient cycling in soil (Chambers et al., 2000; Brown, 2002; Weedon et al., 2009; van Geffen et al., 2010). Fungi are the major decomposers of wood debris, which is an important fraction of an organic matter in forest ecosystems (Owens et al., 1994). Therefore, a lot of attention has been given to community dynamics and succession of fungi during wood decay (Boddy and Watkinson, 1995; Boddy, 2001; Rajala et al., 2011; Fackler and Schwanninger, 2012). However, fungi are not the only microbial inhabitants of decaying wood. Several studies have addressed the occurrence of bacteria in decaying wood and indicated that interactions between fungi and bacteria may be important for the decay processes (de Boer and van der Wal, 2008; Valášková et al., 2009).

Wood-decay fungi can be classified according to the type of decay that they cause namely white- or brown-rot. White-rot fungi are able to degrade lignin in order to get access to other polysaccharides within woody material whereas brown-rot fungi are specialized in degradation of cellulose and hemicellulose without previous lignin removal (Owens et al., 1994). The degradation of wood polymers by fungi is a complex process involving enzymes, mediators, and acidic conditions. Reactive oxygen species that are generated by fungal peroxidases and phenol oxidases have an important role in the degradation of lignin and cellulose. All together, these processes create a harsh environment for bacterial colonization.

The drastic drop of pH upon wood block colonization by the white-rot fungus *Hypholoma fasciculare* had a deleterious and selective effect on wood inhabiting bacteria (de Boer et al., 2010). In contrast, bacterial abundance was high in natural wood samples under decay by *H. fasciculare* despite high acidity and high activity of enzymes producing radical oxygen species (Valášková et al., 2009). Therefore, those bacteria must have been adapted to acidic conditions and oxidative stress (de Boer and van der Wal, 2008). Moreover, in the stages characterized by declining fungal activity (late stage of decomposition), bacteria may become more abundant by being specialized in the degradation of derivatives of lignin decomposition, mainly aromatic compounds.

Whereas, bacteria may have a negative effect on fungal community during wood degradation by competing for sugars released by the fungal extra-cellular enzymes, synergistic effects may also occur (de Boer and van der Wal, 2008). For example, bacteria can provide fungi with limiting nutrients such as iron and nitrogen via nitrogen-fixation (Brunner and Kimmins, 2003; de Boer and van der Wal, 2008; Hoppe et al., 2014) or growth factors like vitamins in exchange for part of easily accessible and degradable carbon sources released by fungal enzymes. It has been shown in some cases that bacteria–fungal consortia are able to degrade wood blocks more effectively than fungi alone (Murray and Woodward, 2003). However, the bacterial and fungal community assembly rules during wood decay are not yet well-studied.

Theories on microbial community assembly have been raised, including the “neutral theory” and the “niche theory” (Dumbrell et al., 2010). The neutral theory predicts that microbial community assembly is a stochastic random process as many species are functionally equivalent in their ability to exploit niches. Thus, their abundance will follow a zero-sum multinomial (ZSM) distribution (Hubbell, 2001; McGill et al., 2006). The niche theory predicts that the microbial community is shaped by abiotic and biotic factors, suggesting that species have unique properties to exploit unique, available niches. Their species abundances will follow pre-emption, broken stick, log-normal, and Zipf-Mandelbrot models (Macarthur, 1957; McGill et al., 2007). Furthermore, these theories suggest that a microbial community driven by environmental parameters would present a deterministic process of assembly. Here, complex interactions, both positive and negative, between bacterial and fungal communities in decaying wood were expected. To gain insight in the importance of bacteria for fungal wood decay, we focused our study on bacterial community composition in relation to changes in fungal community composition during successive stages of natural decay of pine wood. We hypothesized that both the stage of wood decay and changes in the fungal community composition during progressive fungal succession will have an impact on the bacterial community structure. Since, it is expected that one or few fungi are dominant in a decaying unit (van der Wal et al., 2015) we expect a strong selection of bacteria mediated by the dominant fungal species (Folman et al., 2008).

In the current study, we determined the composition and assembly of both bacterial and fungal communities during the stages of natural decay of pine wood. Our primary aim was to determine if dominant wood-decaying fungi drive the dynamics and composition of decaying wood-inhabiting bacterial communities.

## Materials and Methods

### Site Description, Wood Sampling, and Wood Characteristics

Wood samples were collected in autumn 2013 in a mixed forest located near Wolfheze village, the Netherlands (51°59′39″N; 5°47′39″E). Twenty samples of pine wood (*Pinus sylvestris*) were collected from fallen or standing dead tree trunks with different wood density. Wood density was furthermore used as proxy of wood decay stages. For all samples, the bark was removed and slices of wood were surface sterilized under UV light for 30 min. Saw dust was produced by drilling with a sterile drill. Highly decayed wood samples, which could not be drilled, were fragmented using sterile forceps and scalpel. Gravimetric content was determined after drying for 4 days at 60°C. Dried wood was ground with liquid nitrogen in a mortar and carbon and nitrogen content were measured on a Flash EA1112 CN analyzer (Interscience, Breda, the Netherlands). Water extracts of saw dust were prepared by shaking 0.3 g of fresh saw dust with 6 ml milli-Q water at 300 rpm for 1 h. Water extracts were used to determine pH. Ergosterol content was determined as an indication for fungal biomass with alkaline extraction and HPLC analysis (Bååth, 2001). The wood density was established using water-displacement method (Olesen, 1971) allowing measurement for irregularly shaped samples.

### DNA Extraction and PCR Amplification

Wood samples were taken using a sterile wood drill. Four wood dust samples were collected per wood block for separate DNA isolation per replicate. Wood dust was ground in liquid nitrogen. Total genomic DNA was extracted from 150 mg wet weight wood dust using MoBio PowerSoil^TM^ DNA Isolation Kit (MoBio Laboratories, Inc.) according to the manufacturer’s instructions.

Bacterial 16S rRNA V4 gene region was amplified using primer 515f [5′-CCATCTCATCCCTGCGTGTCTCCGACTCAG (MID-10 bases) GTGTGCCAGCMGCCGCGGTAA-3′] constituted of Roche 454 adaptor, 10 bp Roche MID bar-codes and bacterial primer 515f, and reverse primer 806r (5′-CCTATCCCCTGTGTGCCTTGGCAGTCTCAGGGACTACVSGGGTATCTAAT-3′) containing the 454 Life Sciences primer B and the bacterial primer 806r. Internal transcribed spacer region (ITS2) fungal ribosomal operon was amplified using primer ITS9f [5′-CCATCTCATCCCTGCGTGTCTCCGACTCAG (MID-8 bases) GAACGCAGCRAAIIGYGA-3′] and ITS4r (5′-CCTATCCCCTGTGTGCCTTGGCAGTCTCAGCTTCCTCCGCTTATTGATATGC-3′). PCR reactions contained in 25 μl: 1 μl template DNA, 5 μM of each barcoded forward and reverse primer, 2 mM dNTP’s, 1 U FastStart Expand *Taq* DNA polymerase (Roche), and 2.5 μl 10X PCR buffer. The PCR reactions were done under following conditions: initial denaturation step 5 min at 95°C followed by 30 cycles of denaturation for 30 s at 95°C, annealing at 53°C for bacterial 16S rRNA and 58°C for the ITS region for 30 s and extension at 72°C for 1 min. The final extension was extended to 10 min at 72°C. PCR products were purified using the Qiaquick PCR Purification kit (Qiagen). One library for 16S rRNA and one for the ITS region were generated by pooling 80 purified PCR products in equal quantities. Both 16S rRNA and ITS samples were subjected to pyrosequencing on a 454 Life Sciences Genome Sequencer FLX (Roche) machine by Macrogen, Inc. (Seoul, South Korea).

### Pyrosequencing Data Processing

The 16S rRNA data analysis was conducted in Mothur following the standard procedure (Schloss et al., 2009). Briefly, sequences were filtered removing reads that did not have a perfect match to the degenerated primers or barcodes, as well as read that had ambiguous bases and more than six homopolymers. Denoising was done using PyroNoise. Sequences were aligned against reference alignment Silva (Quast et al., 2013). Potential chimeras were identified using the UCHIME program (Edgar et al., 2011) and removed. Filtered sequences were binned into operational taxonomic units (OTUs) based on a 97% dissimilarity cutoff from the distance matrix. One representative sequence of each OTU was assigned through a hierarchical taxonomic annotation using RDP classifier (Wang et al., 2007). For OTU-based analysis, samples were rarefied to 1,080 sequences per sample.

The ITS region sequences were filtered removing reads that did not have perfect match to the primers or barcodes, or that had ambiguous bases and more than eight homopolymers. Denoising was accomplished using PyroNoise. Minimal accepted sequence length was set to 200 bp. Sequences were assigned to the taxonomy in the UNITE v.6.0 database (Kõljalg et al., 2013). The assignment was based on the k-Nearest Neighbor algorithm using a cutoff of 80%. ITS samples were rarefied to 2,000 sequences per sample. The sequencing data are available under accession number PRJEB10643 at European Nucleotide Archive (ENA).

### Statistical Analysis

Correlations between bacterial and fungal community richness and diversity in relation to wood density (proxy of wood decay stage) were calculated using linear regression models. The rarefied OTU table (bacterial community composition) and wood characteristics (pH, moisture, density, nitrogen, carbon, C/N, and ergosterol) data were used for multivariate regression tree (MRT) analysis by using the ‘mvpart’ (Multivariate partitioning) package (De’ath, 2007) in R (statistical programming environment), and the distance matrix was based on Bray–Curtis built by the function “gdist.” Based on MRT results, the samples were classified into three categories: early, middle and late stages.

Significant differences in relative abundance (average value of three replicates) of specific bacterial genera (Supplementary Table S2) and orders (Supplementary Table S3) between categories (early *versus* middle, middle *versus* late and early *versus* late) were determined using Metastats (White et al., 2009). Permutations (1000 bootstrap) were applied for estimating the null distribution of the t statistic. In case of the comparison on the genus taxonomical level, false discovery rate (FDR) control for correcting for multiple comparisons was performed in Metastats. Differences between decay stages were analyzed by one-way ANOVA. *Post hoc* Spjøtfoll–Stoline tests (HSD Tukey for unequal replication numbers) was performed to determine significant differences.

For network analysis OTUs were classified at the genus level (both bacterial and fungal). Only genera with five or more representatives across samples were included in the analysis. The correlation matrices of all pairwise Pearson correlations on bacteria and fungi genus level in different categories (early, middle, and late) and between all samples (average value of three replicates) across three wood samples categories were calculated using R. FDR correcting for multiple comparisons was performed according to Hochberg and Benjamini’s method. Only Genera with the correlation estimate <-0.80 or >0.80 and *P*-value < 0.05 were included in the network analysis. Microbial networks were visualized using Cytoscape (Smoot et al., 2011).

To test whether neutral or niche-based mechanisms best explained the assembly of the microbial community, we examined the species rank abundance distribution for each wood sample category defined by MRT analysis. Niche-based theory assumes that the rank abundance distribution would fit the pre-emption, broken stick, log-normal and Zipf-Mandelbrot models (Motomura, 1932; McGill et al., 2007). On the other hand, the neutral theory predicts that rank abundance distribution would be consistent with ZSM model (Hubbell, 2001). The species rank abundance of each sample were fit to broken stick, pre-emption, log-normal, Zipf, and Zipf-Mandelbrot rank abundance models using the command “radfit” found in the R package vegan (Oksanen et al., 2013), and the ZSM model using TeTame (Jabot et al., 2008). Firstly, we generated the Akaike Information Criterion (AIC), which is a measure of relative quality of a statistical model, providing a means for model selection. These values were calculated based on the equation AIC = -2log-likehood+2^∗^npar, where npar represents the number of parameters in the fitted model (Feinstein and Blackwood, 2012). In order to compare the models, we calculated the Akaike weight (W_i_), which are the weight of evidence in favor of one model being the actual best model in comparison to other tested models (Burnham and Anderson, 2002). The Akaike weight was calculated from the equation W_i_ = exp(-1/2^∗^ΔAIC_i_)/[sum for all model of exp(-1/2^∗^ΔAIC)], where ΔAIC_i_ = AIC_i_ – min(AIC).

## Results

### Diversity and Taxonomic Richness of Bacterial and Fungal Communities in Different Stages of Pine Wood Decomposition

Pine (*Pinus sylvestris*) wood samples were collected based on visual examination of the degree of decomposition. In total, 20 wood samples were collected. Classification of wood samples into the categories early, middle, and late wood decay stages was based on wood density decrease indicating progressive decay. These stages were reflected in shifts in bacterial community structures (for more details see below).

All wood samples were acidic with a significant drop in pH from early to middle and late stages (**Table 1**). Wood density was negatively correlated with water content (*R*^2^ = -0.65, *P* < 0.001). No significant correlations were observed for wood density *versus* C/N ratio or ergosterol content (*R*^2^ = 0.18, *P* = 0.08 and *R*^2^ = 0.13, *P* = 0.13, respectively). The details on physical–chemical characteristics of each of the sample are presented in Supplementary Table S1.

**Table 1:** Wood sample characteristics.

Stage	pH	Relative moisture (%)^∗#^	Wood density [g/cm^3^]	C/N ratio^∗^	Ergosterol [mg/kg]^∗^
Early	4.90^a^	31.62 ± 1.27^b^	0.447 ± 0.027^a^	1009.25 ± 267.19^a^	14.19 ± 1.10^b^
Middle	3.96^b^	33.34 ± 1.24^b^	0.357 ± 0.009^b^	743.02 ± 38.06^ab^	41.98 ± 1.15^a^
Late	4.16^b^	138.99 ± 1.24^a^	0.195 ± 0.0174^c^	267.30 ± 125.34^b^	46.13 ± 1.32^a^

*Mean values and standard error of parameters for each of the wood decay stages (early *n* = 3; middle *n* = 7; late *n* = 8). Differences between decay stages were analyzed by one-way ANOVA. *Post hoc* Spjøtvoll–Stoline tests (HSD Tukey for unequal replication numbers) was performed to determine significant differences.*

*^∗^ANOVA and *post hoc* test performed after log-transformation.*

*^#^Moisture [%] = (weight of water/oven dry weight of wood) × 100.*

*Values with the same letters (a, b, c) were not significantly different (*P* < 0.05).*

From the wood samples, a total of 523,587 high quality bacterial and 1,304,635 high quality fungal sequences were obtained. The number of reads per sample varied between 300 (sample Pi03) and 19,553 for bacterial samples, and between 2,111 and 39,245 for fungal samples. Sample Pi03 was excluded from the analysis due to amplification bias and low yield of sequence reads for both the 16S rRNA and ITS regions. For the same reason, sample Pi02 was excluded from bacterial based analysis. Both excluded samples belonged to the early stage of decay (dead tree trunk, high wood density, low water content, no discoloration).

In order to retrieve higher number of reads per sample, three out of four replicates per sample (the replicate with lowest read number was discarded) were analyzed. To perform the analysis, sequences were rarefied to 1,080 reads per sample (in total 72,360 reads) for bacterial samples and to 2,000 reads per sample (in total 144,000 reads) for fungal samples.

Cluster analysis at 97% cutoff identified 1,065 bacterial OTUs across samples with 208 OTUs comprising single sequences. The number of OTUs per sample varied between 35 for sample Pi01 with the highest wood density (0.5 g/cm^3^), and 237 for sample Pi17 in the late stage of decomposition and wood density of 0.19 g/cm^3^.

**FIGURE 1:**
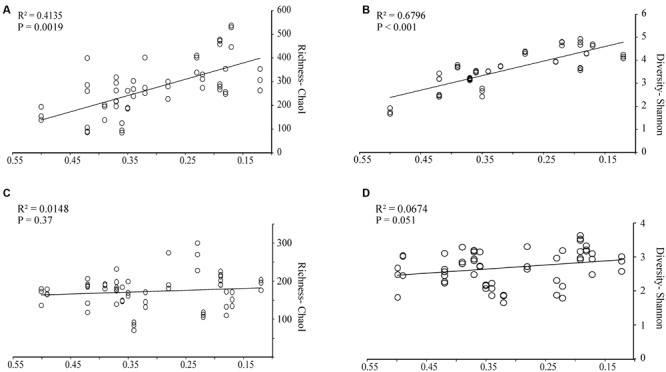
**Bacterial and fungal community richness and diversities in relation to wood decay stages as based on wood density.** Linear regression with **(A)** bacterial OTU richness (Chao1 estimator), **(B)** bacterial diversity (Shannon index), **(C)** fungal OTU richness (Chao1 estimator), and **(D)** fungal diversity (Shannon index). The line represents the best-fit linear regression model to the dataset. The indexes were calculated using 1,080 reads per sample for 16S rRNA and 2,000 reads per sample for ITS region, respectively. Three replicate measurements per wood sample are included.

Regression analyses were performed for alpha diversity measurements of bacterial and fungal OTUs *versus* wood characteristics (**Figure 1**). For the bacterial communities, species richness, expressed as the ChaoI estimator or the Shannon index, correlated positively and significantly (*P* < 0.001 and *P* = 0.0019, respectively) with the progress of wood-decay (**Figure 1**). In both cases the decay stage accounted for approximately half of the variability of the samples, with *R*^2^ = 0.41 for ChaoI estimator and *R*^2^ = 0.67 for Shannon diversity index *versus* wood density, respectively. There were no significant correlation between alpha diversity measurements and wood pH, C/N ratio or ergosterol-based fungal biomass (data not shown). For the fungal ITS region, OTUs were defined based on counts of unique sequences. Contrary to the bacterial communities, neither fungal sequence richness (ChaoI estimator) nor diversity (Shannon index) was correlated with wood density loss (**Figures 1C,D**, respectively).

**FIGURE 2:**
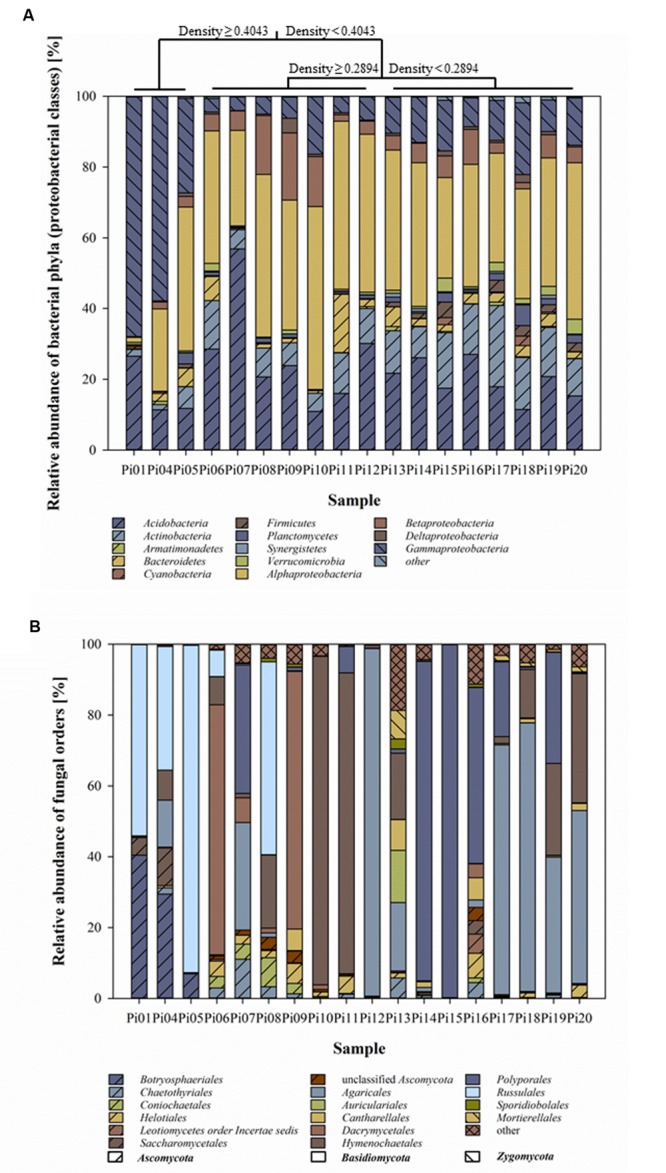
**Bacterial **(A)** and fungal **(B)** community compositions in decaying pine-wood presented as relative abundance of taxa at the phylum level for bacteria (class level for *Proteobacteria*) and at the order level for fungi.** Bar-plots show the average abundance of three replicates per sample. Wood samples were organized according to MRT analysis based on OTUs identified in rarefied to 1,080 reads per replicate for bacterial 16S rRNA and to 2,000 for fungal ITS region per sample and wood characteristics. Taxonomic groups representing low abundance (<5% of total) are grouped and presented under the name “other.”

The taxonomic affiliations of the bacterial reads are given in **Figure 2A**. Next to the dominant phylum *Proteobacteria*, especially *Alpha*- and *Gamma-Proteobacteria*, a high proportion of reads was classified as *Acidobacteria* ranging from 11 up to 57% (average 22%). The majority of acidobacterial sequences were affiliated with subdivisions 1 and 3, however, subdivisions 2, 4, 6, 7, 14, and 16 were also identified. Fungal community compositions almost entirely consisted of *Basidio*- and *Ascomycota* with 95–100% of sequences assigned to those two phyla, respectively. Each of the samples had its own fungal community signature. As expected, communities were mostly dominated by one fungal phylotype (one sequence type; **Figure 2B**).

### Bacterial Community in Relation to Wood Characteristics and Fungal Communities

Multivariate regression tree analyses classified the wood samples based on wood density (characteristic that reflects wood decay) into three clusters, namely: early, middle, and late wood decay stages. Samples with wood density above 0.40 g/cm^3^ were classified as early stage (samples Pi01, Pi04, and Pi05). Samples with wood density between 0.40 and 0.30 g/cm^3^ were classified as middle (samples Pi06, Pi07, Pi08, Pi09, Pi10, Pi11, and Pi12) and samples with wood density below 0.30 g/cm^3^ as late wood decay stage (samples Pi13, Pi14, Pi15, Pi16, Pi17, Pi18, Pi19, and Pi20; **Figure 2A**).

The average values for each of the wood properties in the three decay stages are given in **Table 1**. The pH significantly dropped (*P* < 0.01) from early (4.90 ± 0.12) to middle (3.96 ± 0.08) and late stages (4.16 ± 0.14). The relative wood moisture (%) increased significantly at late stage: 33.62 ± 1.27 in early, 33.34 ± 1.24 in middle and 138.99 ± 1.24 in late stages (*P* < 0.010). The C/N ratio was significantly lower *P* < 0.05) at late wood decay stage (267.30 ± 125.34) as compared to early (1009.25 ± 267.19) and middle (743.02 ± 38.06) stages. Ergosterol content significantly increased at middle and late stages *P* < 0.03).

**FIGURE 3:**
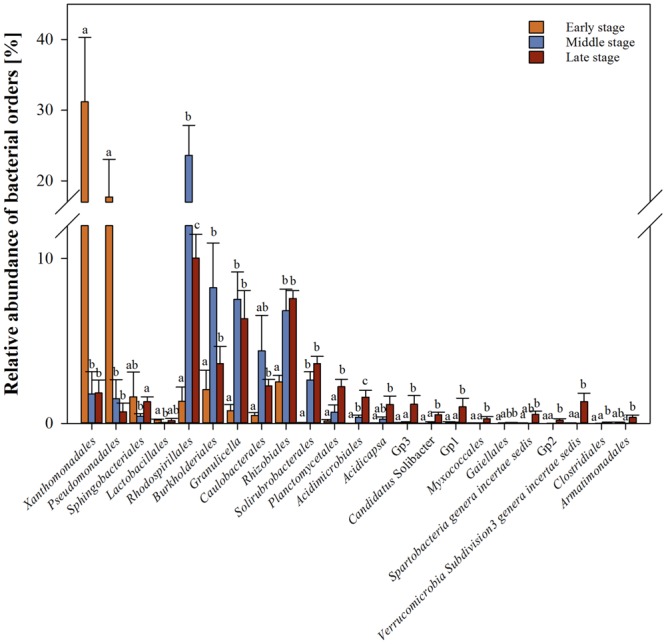
**Bacterial Orders differentially represented between wood decay stages.** Comparisons are shown only for orders with significant differences (*P* < 0.05). Bars represent standard errors.

In total, 18 bacterial genera significantly (*q*-value ≤ 0.05) differed in their abundance between early and middle stages of wood decay, 32 between middle and late and, 28 between early and late stages (Supplementary Table S2). To simplify the output, the analysis was performed on the Order taxonomical level (**Figure 3** and Supplementary Table S3). The early stage was overrepresented by sequences classified as *Gammaproteobacteria* especially those affiliated with the Order *Xanthomonadales* (31 in early and 2% in middle and late stages, respectively) and *Pseudomonadales* (18 in early and 1% in middle and late stages, respectively). In the middle stage, the orders affiliated with *Alphaproteobacteria; Rhodospirillales* (24%) and with *Betaproteobacteria; Burkholderiales* (8%) were overrepresented.

**FIGURE 4:**
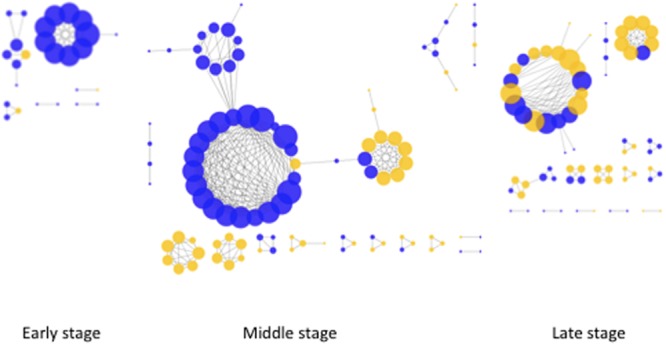
**Network co-occurrence analysis of bacterial and fungal community compositions within different stages of wood decay.** Analyses were performed at the order level. Positive correlation (with estimate value > 0.8) is indicated as continues edges. Only correlations with adjusted *P*-value < 0.001 are presented. The size of the node corresponds to the number of connections. Blue nodes are bacterial and yellow are fungal Orders.

In order to test how the relationship between microorganisms (microbial and fungal taxa) shape the bacterial community structure, we performed network interference analysis. Co-occurrence analysis were performed for each of the decay stages separately (**Figure 4**). The highest number of correlations were observed in middle stage followed by late and early decay stages (100, 71, and 26 nodes, respectively). The same order was followed by the average degree of node connectivity (5.76, 4.03, and 4.00). Overall we could discern increased complexity of the relations in the middle stage of decay. Interestingly, in the early and middle stages only single bacteria–fungi co-occurrence correlations were observed. Taking into account all bacteria and fungi samples for a general co-occurrence analysis (Supplementary Figure S1), the location of the genera in the network was evaluated based on the degree of centrality. Degree of centrality defined key nodes (in this case genera) in the network with the highest number of correlations to other taxa in the network. The majority of genera identified as a key nodes were affiliated with *Acidobacteria, Actinobacteria*, and *Proteobacteri*a (Supplementary Table S4). Overall, bacterial and fungal communities shared low connectivity. Out of 121 bacterial genera entering the network, only 14 showed direct connections with fungal genera (Supplementary Table S5). The majority of direct bacterial fungal connections were observed between *Acidobacteria* especially belonging to subdivision 1.

### Neutral or Niche-Based Assembly Predictions

**FIGURE 5:**
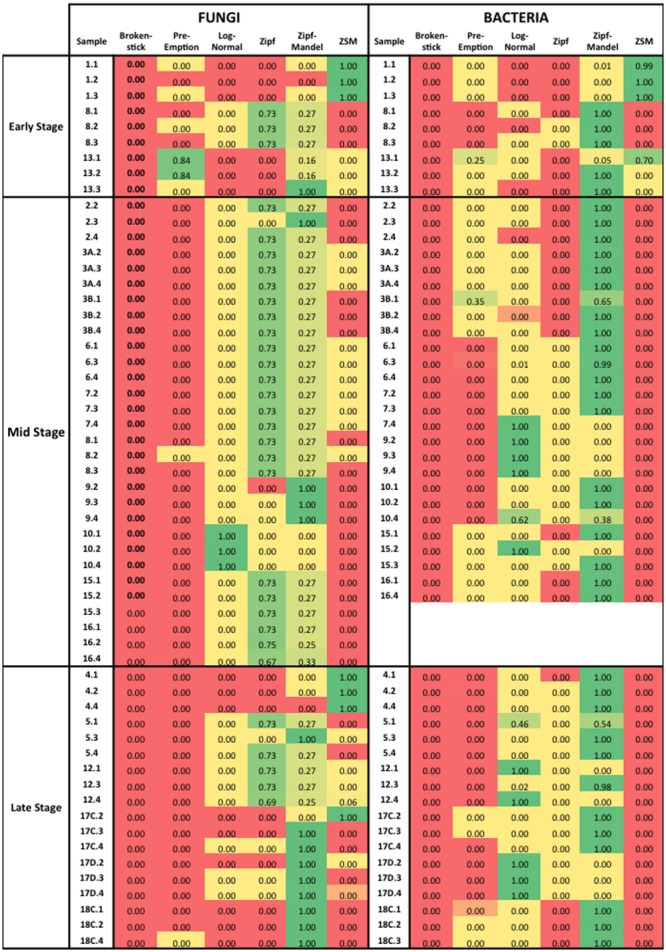
**Akaike Information Criterion (AIC) weight values for six rank abundance distribution models used in this work.** The AIC weight varies from 0 to 1, being the highest value the best-fit model. The color scale was used for a better visualization, where green indicates the best model.

The species abundance distribution of all samples was fitted to six theoretical models to test whether neutral or niche-based mechanisms best explained microbial community assembly patterns in the wood samples from three degrees of decomposition (Supplementary Tables S5–S11). The choice for the best model was made based on the comparison of the AIC weight, as indicated in the **Figure 5**. The comparison of different rank abundance distribution models based on AIC weight values indicated that at the early stage, the data fitted both neutral and niche-based models. This result indicates stochastic mechanisms in the assembly of the community at the early stage of the wood decomposition. In other words, the composition of the microbial community at early stage is based on aleatory mechanisms, without a selection of specific groups based on niche availability. However, in the middle and late stages, niche-based mechanisms best explained microbial community assembly, which indicates that species are selected based on their ability to inhabit and exploit new niches available during the decomposition of the wood. This is an indication that environmental parameters, such as chemical properties of the wood, are driving the community composition by selecting specific microbial groups.

## Discussion

The relationship between bacterial and fungal composition during wood decay is not yet well-studied. In our study, we focused on microbial community development during the decomposition of wood of the tree species *Pinus sylvestris*. We used wood density as a proxy for wood decay stage. However, it must be noted that the bacterial and fungal communities development may vary between different wood samples as a result of differences in the initial colonization.

The shift in bacterial communities in response to progressive decomposition was reflected by a positive correlation between bacterial richness and diversity *versus* the stage of wood decay. Interestingly, bacterial community diversity as well as richness, though strongly correlated with the stage of decay, was not linearly correlated with C/N ratio. Decaying wood is considered to have a high C/N ratio and thus may be a difficult substrate to degrade under N limitation. Furthermore, all analyzed samples, despite the stage of decay, showed high C/N ratios, suggesting nitrogen was a limiting factor for degradation. However, an increase in total N content with progressive decomposition was observed in the samples. The increase in N content may be related with microbial activities as have been shown for some fungi and bacteria. For instance, *H. fasciculare* was recently shown to be able to translocate N into decomposing wood from soil under the colonized wood (Philpott et al., 2014). Similarly, wood-inhabiting N-fixing bacteria were suggested to support fungi in fulfilling their N requirements (Cowling and Merrill, 1966; Hoppe et al., 2014). The identification of potential N-fixing bacteria in our study is discussed below.

The bacterial communities were dominated by *Acidobacteria, Alpha*-, *Beta*-, *Gammaproteobacteria, Firmicutes*, and *Actinobacteria*. The dominance of those phyla in decaying wood was reported before (e.g., Zhang et al., 2008; Valášková et al., 2009). Here, the early stage was dominated by two groups of bacteria, namely *Xanthomonadales* and *Pseudomonadales*. These bacteria are known to be fast growing bacterial groups and to be metabolically highly versatile. Members of the first group include phytopathogens that cause a wide variety of serious plant diseases. *Pseudomonadales* are affiliated with plant pathogenic bacteria as well but also described as a group of endophytic and plant beneficial bacteria. Bacteria belonging to those two groups were also previously reported as being associated with the pinewood nematode, *Bursaphelenchus xylophilus*, causal agent of the Pine Wilt Disease (Proença et al., 2010; Tian et al., 2011; Vicente et al., 2011) and with wood-feeding beetle larvae (*Prionoplus reticularis*; Reid et al., 2011). *Xanthomonadaceae* were previously shown to be dominant among isolates obtained from beech wood (Folman et al., 2008; Valášková et al., 2009) and wood sawdust inoculated with *Phanerochaete chrysosporium* (Hervé et al., 2014). *Pseudomonas* and *Luteibacter*, two of the most abundant genera, were identified as dominant in freshly cut pine wood chips (Noll et al., 2010).

Interestingly, we observed high relative abundances of *Acidobacteria*, especially subdivisions 1 and 3. The predominance of *Acidobacteria* in low pH conditions (such as decaying wood) is well-documented for members of subdivision 1 (Sait et al., 2006; Jones et al., 2009). There are also isolates of this subdivision that were previously obtained from wood (Valášková et al., 2009; Yamada et al., 2014). Supplementation of growth media with plant polymers has been suggested as a method to increase cultivability of *Acidobacteria* subdivisions 1 and 3 (Eichorst et al., 2011). Up to date, only two recognized and described acidobacterial species were obtained from wood: *Acidicapsa ligni* isolated from wood samples in advanced stage of decay and colonized by *H. fasciculare* (Valášková et al., 2009) and *Granulicella cerasi* isolated from bark of a living cherry tree (Yamada et al., 2014). There are no reports on the potential function of *Acidobacteria* in decomposition processes nor on potential interaction with fungi. It may be only speculated based on their described characteristics (Vorob’ev et al., 2009; Yamada et al., 2014) that these bacteria are well-adapted to wood decay condition. They may interact with fungi as both isolates are able to grow on trehalose, a disaccharide used for carbon storage by fungi. Additionally, *A. ligni* (no information available for *G. cerasi*) is able to grow on oxalate, a substrate that would be highly available as exudate of wood decomposing fungi.

The number of bacterial groups known to be associated with fungal activity and remove toxic wood compounds, thus exerting an expected positive effect on fungal communities, increased in the middle (e.g., *Burkholderia, Phenylobacterium, Acidisoma*) or in middle and late decay stages (*Methylovirgula*). Bacteria belonging to *Burkholderiales* have been shown to be able to degrade cellulose, having oxalotrophic activities but also being able to degrade aromatic compounds (Sahin, 2003; Bugg et al., 2011; Eichorst and Kuske, 2012; Štursová et al., 2012). Also, *Phenylobacterium* may be involved in removal of toxic aromatic compounds from wood (Lingens et al., 1985). Another interesting group of microorganisms abundant in middle and late stages is the genus *Methylovirgula.* These bacteria use methanol produced during wood decomposition as carbon source. Additionally they can utilize oxalate but also are capable of atmospheric nitrogen fixation (Vorob’ev et al., 2009). As mentioned before, due to low N availability in the decaying wood it was hypothesized that fungi may be supported by N-fixing bacteria (Cowling and Merrill, 1966). Although, we identified potential N-fixing candidates (*Rhizobiales, Methylovirgula*) as more abundant in middle and late stages than early stage, these two groups were not dominant in the microbial communities.

Although, the fungal community compositions were highly variable (**Figure 2B**) between wood samples, bacterial communities were much less variable (**Figure 2A**). Also, we were able to group samples according to the bacterial community composition and wood-decay stages (wood density loss) into three decay stages. It was surprising that the bacterial communities were not connected with the fungal species present in the wood. The co-occurrence analysis showed low dependence (low connectivity) in early and middle stages of decay between bacteria and fungi. The analysis indicated that bacteria responded more to the changes in wood physicochemical properties as a result of fungal driven decomposition rather than to the cause of those changes, fungal species.

In order to test the role of changes in the physicochemical parameters of the wood on the community composition, we fit species rank abundances to assembly models. In the early stage of wood decay, our results showed that both bacterial and fungal genera distributions indicate the relevance of stochastic processes (Hubbell, 2001; McGill et al., 2006). In some systems, both deterministic and stochastic processes are responsible for constructing ecological communities (Chave, 2004). At the middle stage, all the samples fitted niche-based models. At the late stage, all bacteria samples fitted niche-models, while for fungi there was also a contribution of stochastic process. These results pointed to a microbial community selection via niche filtering, mainly at the middle stage of the wood decomposition. The selected groups of bacteria of very low abundance (some of those were specific to the decay stage) may require specific nutritional conditions although close dependence/interaction with fungi cannot be excluded. This hypothesis is supported by the species abundance distribution in the middle and late decay stages for which niche-based mechanisms better explained the microbial community assembly. Niche theory predicts that changes in species composition are not random and are related to changes in environmental variables. In our study, both bacterial and fungal communities could be fitted to the niche theory models at the middle stage of wood decomposition. Although, stage of decomposition had an effect on shaping the overall microbial community, the identification of the major cause among environmental factors is more complex as multiple factors co-vary and only a limited number of parameters was measured in this study. This may therefore be a good avenue for future research. In case of fungal communities, competitive interactions between fungi may have a strong and deterministic impact on the community composition. The succession type of fungal community shifts during wood degradation is well-documented (Rajala et al., 2011; Fackler and Schwanninger, 2012).

In our study, we focused on the bacterial communities in the process of wood decay. We showed that (1) bacterial communities underwent less drastic changes compared to the fungal communities; (2) the community assemblage in the early stage was a stochastic process; over wood decay progression, the community was determined by environmental factors; (3) bacteria community composition responded to the stage of decay (reflected by the wood density) more than fungal community composition. Thus, we conclude that the changing conditions and wood properties as a result of fungal activity were more important determinants of bacterial community composition than the taxonomic classification of those fungi. This finding is also supported by the low number of co-occurrence correlations between bacterial and fungal community members.

## Author Contributions

AK and TS designed the study, collected all data and conducted the analyses. AK wrote the manuscript with assistance of EK. LM and EK contributed to the analyses and to the revision of the final manuscript. JV and EK provided conceptual input and contributed to the critical revision of the manuscript. All authors approved the final version of the manuscript.

## Conflict of Interest Statement

The authors declare that the research was conducted in the absence of any commercial or financial relationships that could be construed as a potential conflict of interest.
