# Supplementary material for: Bacterial Community Succession in Pine-Wood Decomposition
Source: Front Microbiol. 2016 Mar 1;7:231. doi: 10.3389/fmicb.2016.00231 (PMC4771932; doi:10.3389/fmicb.2016.00231)
Supplement: Supplementary file 1 [file Data_Sheet_1.DOCX]

**Supplementary material**

**Bacterial community succession in pine-wood decomposition**

Anna M. Kielak, Tanja R. Scheublin, Lucas W. Mendes, Johannes A. van Veen, Eiko E. Kuramae

**Table S1. Wood samples characteristics**

| **Sample** | **pH** | **Moisture*** | **Density** | **Nitrogen** | **Carbon** | | **C/N** | | **Ergosterol** | | |
| --- | --- | --- | --- | --- | --- | --- | --- | --- | --- | --- | --- |
|  |  | [%] | [g/cm3] | [%] | [%] | |  | | [mg/kg dry wood] | | |
| Pi01 | 5.1 | 22.5 | 0.50 | 0.07 | 49 | | 674 | | 12 | | |
| Pi02 | 4.0 | 18.9 | 0.49 | 0.05 | 47 | | 963 | | 37 | | |
| Pi03 | 4.3 | 19.1 | 0.45 | 0.02 | 51 | | 2384 | | 1 | | |
| Pi04 | 4.9 | 28.1 | 0.42 | 0.05 | 47 | | 911 | | 14 | | |
| Pi05 | 4.7 | 49.9 | 0.42 | 0.03 | 47 | | 1421 | | 17 | | |
| Pi06 | 4.1 | 20.6 | 0.39 | 0.07 | 48 | | 653 | | 60 | | |
| Pi07 | 3.7 | 25.5 | 0.37 | 0.05 | 48 | | 1028 | | 55 | | |
| Pi08 | 4.2 | 22.1 | 0.37 | 0.07 | 47 | | 653 | | 29 | | |
| Pi09 | 4.0 | 27.9 | 0.36 | 0.06 | 47 | | 756 | | 62 | | |
| Pi10 | 4.2 | 25.4 | 0.35 | 0.06 | 47 | | 734 | | 28 | | |
| Pi11 | 3.7 | 74.0 | 0.34 | 0.07 | 48 | | 678 | | 49 | | |
| Pi12 | 3.8 | 75.5 | 0.32 | 0.07 | 47 | | 665 | | 28 | | |
| Pi13 | 4.0 | 131.1 | 0.28 | 0.11 | 48 | | 431 | | 28 | | |
| Pi14 | 4.0 | 145.9 | 0.23 | 0.22 | 52 | | 243 | | 44 | | |
| Pi15 | 3.7 | 57.6 | 0.22 | 0.02 | 46 | | 2143 | | 51 | | |
| Pi16 | 3.9 | 60.1 | 0.19 | 0.10 | 47 | | 481 | | 57 | | |
| Pi17 | 4.4 | 128.5 | 0.19 | 0.44 | 48 | | 108 | | 207 | | |
| Pi18 | 4.6 | 251.0 | 0.18 | 0.19 | 44 | | 230 | | 13 | | |
| Pi19 | 4.8 | 237.1 | 0.15 | 0.41 | 50 | | 125 | | 57 | | |
| Pi20 | 3.9 | 273.8 | 0.12 | 0.66 | 50 | | 75 | | 37 | | |
| * Moisture [%] = (weight of water / oven dry weight of wood) x 100. | | | | | |  | |  | |  |  |

**Table S2. Differences in relative abundance of bacterial genera between different stages of wood decay. Mean values (% of total count) and standard deviation of each of the differentially abundant genera. Letters indicate differences in abundance between different stages of decay**

**(q < 0.05).**

| phylum | class | order | family | genus | early | middle | late |
| --- | --- | --- | --- | --- | --- | --- | --- |
| Acidobacteria | Gp1 | Acidicapsa | Acidicapsa | Acidicapsa | 0,01±0,01a | 0,36±0,06b | 1,34±0,34b |
|  |  | Gp1 | Gp1 | Gp1 | 0,07±0,062ab | 0,045±0,02a | 1,31±0,38b |
|  |  | Granulicella | Granulicella | Granulicella | 0,81±0,22a | 6,93±0,76b | 6,72±1,16b |
|  |  | Terriglobus | Terriglobus | Terriglobus | 2,17±0,70a | 0,36±0,08a | 0,01b |
|  | Gp2 | Gp2 | Gp2 | Gp2 | 0a | 0a | 0,28±0,09b |
|  | Gp3 | Candidatus Solibacter | Candidatus Solibacter | Candidatus Solibacter | 0,01±0,01a | 0,06±0,03a | 0,68±0,10b |
|  |  | Gp3 | Gp3 | Gp3 | 0,04±0,03a | 0,07±0,02a | 1,53±0,37b |
|  | unclassified | unclassified | unclassified | unclassified | 4,21±1,17a | 14,75±1,18b | 23,52±1,952c |
| Actinobacteria | Actinobacteria | Acidimicrobiales | Acidimicrobineae_incertae_sedis | Aciditerrimonas | 0ab | 0a | 0,04±0,02b |
|  |  |  | Actinomycetaceae | Actinomyces | 0,01±0,01ab | 0,04±0,02a | 0b |
|  |  |  | Corynebacteriaceae | Corynebacterium | 0ab | 0,09±0,04a | 0b |
|  |  |  | Microbacteriaceae | Gryllotalpicola | 0a | 0,46±0,16b | 0,10±0,05ab |
|  |  |  | Mycobacteriaceae | Mycobacterium | 0,91±0,46a | 4,36±0,541b | 1,64±0,42a |
|  |  |  | Pseudonocardiaceae | Actinomycetospora | 0,01±0,01ab | 0,05±0,03a | 0b |
|  |  |  | Streptomycetaceae | Streptacidiphilus | 0a | 0,07±0,07a | 0,13±0,04b |
|  |  | Solirubrobacterales | Conexibacteraceae | Conexibacter | 0,03±0,015a | 1,25±0,19b | 1,84±0,35b |
|  | unclassified | unclassified | unclassified | unclassified | 0,91±0,24a | 6,08±1,20b | 6,10±0,27b |
| Armatimonadetes | Armatimonadia | Armatimonadales | Armatimonadaceae | Armatimonas/  Armatimonadetes_gp1 | 0a | 0,02±0,01a | 0,45±0,12b |
| Bacteroidetes | Sphingobacteriia | Sphingobacteriales | Chitinophagaceae | Ferruginibacter | 0ab | 0a | 0,05±0,02b |
|  | unclassified | unclassified | unclassified | unclassified | 1,11±0,54ab | 0,25±0,05a | 1,13±0,12b |
| Firmicutes | Bacilli | Lactobacillales | Lactobacillaceae | Lactobacillus | 0,07±0,07a | 0b | 0b |
| Planctomycetes | Planctomycetia | Planctomycetales | Planctomycetaceae | Aquisphaera | 0a | 0,22±0,05a | 0,94±0,11b |
|  |  |  |  | Blastopirellula | 0a | 0a | 0,09±0,02b |
|  | unclassified | unclassified | unclassified | unclassified | 0,02±0,01a | 0,63±0,16b | 1,45±0,24b |
| Proteobacteria | α-proteobacteria | Caulobacterales | Caulobacteraceae | Asticcacaulis | 0ab | 0a | 0,06±0,03b |
|  |  |  |  | Brevundimonas | 0,15±0,06a | 0,03±0,02ab | 0b |
|  |  |  |  | Phenylobacterium | 0,19±0,10a | 3,35±0,97b | 1,55±0,20b |
|  |  | Rhizobiales | Beijerinckiaceae | Methylorosula | 0,12±0,05ab | 0,21±0,07a | 0,03±0,02b |
|  |  |  |  | Methylovirgula | 0,07±0,04a | 5,01±0,58b | 4,51±0,43b |
|  |  |  | Hyphomicrobiaceae | Rhizomicrobium | 0a | 0,08±0,04ab | 0,28±0,12b |
|  |  |  | Rhizobiaceae | Rhizobium | 1,06±0,42a | 0,352±0,212ab | 0,012±0,01b |
|  |  | Rhodobacterales | Rhodobacteraceae | Paracoccus | 0,01±0,01ab | 0,06±0,04a | 0b |
|  |  |  | Acetobacteraceae | Acidisoma | 0,56±0,21a | 4,58±0,43b | 1,33±0,20a |
|  |  | Sphingomonadales | Sphingomonadaceae | Sphingomonas | 1,68±0,40a | 0,39±0,08a | 0,01±0,01b |
|  |  | unclassified | unclassified | unclassified | 19,58±5,39ab | 25,67±1,82a | 14,32±0,76b |
|  | β-proteobacteria | Burkholderiales | Alcaligenaceae | Achromobacter | 0,08±0,04a | 0b | 0,01±0,01b |
|  |  |  | Burkholderiaceae | Burkholderia | 1,29±0,36a | 7,98±1,18b | 2,26±0,43a |
|  | γ-proteobacteria | Pseudomonadales | Pseudomonadaceae | Pseudomonas | 18,83±3,32a | 1,68±0,54b | 0,024±0,01c |
|  |  | unclassified | unclassified | unclassified | 0,07±0,05ab | 0,014±0,01a | 0,31±0,09b |
|  |  |  |  |  | 8,89±4,37ab | 1,36±0,27a | 6,79±0,85b |
|  |  | Xanthomonadales | Xanthomonadaceae | Luteibacter | 15,89±1,82a | 0,51±0,19b | 0c |
|  |  |  |  | Pseudoxanthomonas | 7,84±3,33a | 0,01±0,01b | 0b |
|  | unclassified | unclassified | unclassified | unclassified | 0,76±0,23a | 3,80±0,55b | 8,49±0,74c |
| Verrucomicrobia | Spartobacteria | Spartobacteria_genera_incertae_sedis | Spartobacteria_genera_incertae_sedis | Spartobacteria_genera_incertae_sedis | 0,03±0,02a | 0,03±0,01a | 0,68±0,14b |
|  | Subdivision3 | Subdivision3_genera_incertae_sedis | Subdivision3_genera_incertae_sedis | Subdivision3_genera_incertae_sedis | 0,01±0,01a | 0,07±0,05a | 1,72±0,36b |
|  | unclassified | unclassified | unclassified | unclassified | 0ab | 0a | 0,09±0,03b |

**Table S3. Differences in relative abundance of bacterial Orders between different stages of wood decay. Mean values (% of total count) and standard deviation of each of the differentially abundant genera. Letters indicate differences in abundance between different stages of decay**

**(p < 0.05).**

| **Order** | **early** | **middle** | **late** |
| --- | --- | --- | --- |
| **Acidimicrobiales** | 0,49±0,45a | 1,19±0,47ab | 1,88±0,27b |
| **Actinomycetales** | 2,25±0,49a | 5,54±0,89b | 7,18±0,98b |
| **Bacillales** | 0,22±0,17a | 0,27±0,21a | 1,22±0,18b |
| **Bacteroidales** | 0ab | 0a | 0,09±0,04b |
| **Burkholderiales** | 1,73±0,86a | 8,7±2,02b | 2,58±0,68a |
| **Chromatiales** | 0,15±0,11a | 0,43±0,26a | 3,20±1,06b |
| **Edaphobacter** | 0ab | 0a | 0,19±0,08b |
| **Erysipelotrichales** | 0,03±0,03a | 0,04±0,02a | 0,1±0,02b |
| **Flavobacteriales** | 0,06±0,03a | 0,15±0,05ab | 0,37±0,12b |
| **Gammaproteobacteria_incertae_sedis** | 0ab | 0,04±0,03a | 0,26±0,08b |
| **Gp1** | 0,96±0,46a | 8,73±1,73b | 4,87±0,58c |
| **Gp3** | 0,06±0,06a | 2,00±1,00ab | 1,30±0,39b |
| **Haloplasmatales** | 0ab | 0a | 0,09±0,04b |
| **Lactobacillales** | 0,03±0,03ab | 0a | 0,42±0,19b |
| **Legionellales** | 0,03±0,03a | 0,31±0,10b | 1,5±0,29c |
| **Neisseriales** | 0,03±0,03a | 0,18±0,07a | 0,8±0,24b |
| **Pseudomonadales** | 18,41±6,32a | 1,22±0,83b | 0,37±0,09b |
| **Rhizobiales** | 1,11±0,97a | 7,35±1,44b | 7,58±0,89b |
| **Rhodospirillales** | 2,16±1,44a | 20,03±2,34b | 20,60±1,03b |
| **Rickettsiales** | 0,09±0,00a | 0,04±0,04ab | 0,01±0,02b |
| **Solirubrobacterales** | 0,62±0,6a | 3,19±0,75b | 3,37±0,50b |
| **Synergistales** | 0,03±0,03a | 0,11±0,03a | 0,68±0,15b |
| **Xanthomonadales** | 29,65±7,90a | 2,32±0,91b | 5,05±0,78c |

**Table S4.** Top 10 bacterial genera with the highest centrality degree values.

| Taxonomy | degree |
| --- | --- |
| *Acidobacteria;* Gp1*;* unclassified | 40 |
| *Acidobacteria;* Gp2; unclassified | 40 |
| *Armatimonadetes; Armatimonadia; Armatimonadales; Armatimonadaceae; Armatimonas/Armatimonadetes_*gp1 | 17 |
| *Planctomycetes; Planctomycetia; Planctomycetales; Planctomycetaceae; Blastopirellula* | 17 |
| *Proteobacteria; Alphaproteobacteria; Caulobacterales; Caulobacteraceae; Caulobacter* | 13 |
| *Acidobacteria;* Gp1*; Acidicapsa* | 12 |
| *Actinobacteria; Actinobacteria; Actinomycetales; Pseudonocardiaceae; Actinomycetospora* | 12 |
| *Actinobacteria; Actinobacteria; Actinomycetales; Corynebacteriaceae; Corynebacterium* | 12 |
| *Proteobacteria; Alphaproteobacteria; Caulobacterales; Rhodospirillales; Acetobacteraceae; Acidocella* | 11 |

**Table S5.** Bacterial genera with direct connection to fungal genera (positive Pearson correlation).

| **Bacterial Genus** | **Fungal Genus** |
| --- | --- |
| *Acidobacteria*; Gp1 | *Botryobasidium* |
|  | *Cordyceps* |
|  | *Exophiala* |
|  | *Fusarium* |
|  | *Guepinia* |
|  | *Hypocrea* |
|  | *Mortierella* |
|  | *Pluteus* |
|  | *Pochonia* |
|  | *Sporobolomyces* |
|  | Unclassified *Helotiales*43 |
|  | Unclassified *Mortierellaceae*4 |
|  | Unclassified *Mortierellaceae*54 |
|  | Unclassified *Mortierellaceae*87 |
|  | Unclassified *Mortierellaceae*9 |
| *Acidobacteria*; Gp1; *Acidicapsa*; | Unclassified *Mortierellaceae*18 |
|  | Unclassified *Polyporales*1016 |
|  | Unclassified *Polyporales*1051 |
| *Acidobacteria*; Gp2 | Unclassified *Polyporales*1116 |
|  | Unclassified *Polyporales*402 |
|  | Unclassified *Polyporales*433 |
|  | Unclassified *Polyporales*453 |
|  | Unclassified *Polyporales*664 |
|  | Unclassified *Polyporales*735 |
|  | Unclassified *Polyporales*809 |
|  | *Botryobasidium* |
|  | *Cordyceps* |
|  | *Exophiala* |
|  | *Fusarium* |
| *Acidobacterila*; Gp3; *Candidatus*Solibacter  *Actinobacteria*; *Actinobacteria*; *Actinomycetale*i; *Corynebacteriaceae*; *Corynebacterium*  *Actinobacteria*; *Actinobacteria*; *Actinomycetales*; *Pseudonocardiaceae*; *Actinomycetospora* | *Guepinia* |
|  | *Hypochnicium* |
|  | *Hypocrea* |
|  | *Mortierella* |
|  | *Pluteus* |
|  | *Pochonia* |
|  | *Sporobolomyces* |
|  | unclassified_*Helotiales*43 |
|  | unclassified_*Mortierellaceae*3 |
|  | unclassified_*Mortierellaceae*4 |
|  | unclassified_*Mortierellaceae*54 |
|  | unclassified_*Mortierellaceae*87 |
|  | unclassified_*Mortierellaceae*9 |
|  | *Kazachstania* |
|  | *Trichosporon* |
|  | *Ischnoderma* |
|  | *Ischnoderma* |
| *Armatimonadetes*; *Armatimonadetes* gp5 | *Aphanobasidium* |
|  | *Cortinarius* |
|  | *Trichoderma* |
| *Planctomycetes*; *Planctomycetia*; *Planctomycetales*; *Planctomycetaceae*; *Blastopirellula*  *Planctomycetes*; *Planctomycetia*; *Planctomycetales*; *Planctomycetaceae; Gemmata* | unclassified_*Ascomycota*131 |
|  | unclassified_*Mortierellaceae*43 |
|  | unclassified_*Polyporales*1059 |
|  | unclassified_*Polyporales*599 |
|  | unclassified_*Polyporales*671 |
|  | *Hypochnicium* |
|  | *Trichoderma* |
|  | *Xylodon* |
| *Proteobacteria*; *Alphaproteobacteria*; *Caulobacterales*; *Caulobacteraceae; Caulobacter* | *Ischnoderma* |
|  | *Coprinellus* |
| *Proteobacteria*; *Betaproteobacteria*; *Burkholderiales*; *Burkholderiaceae*; *Burkholderia* | *Heterobasidion* |
|  | *Melanchlenus* |
|  | *Mycosphaerella* |
|  | *Postia* |
|  | unclassified_*Polyporales*669 |
|  | unclassified_*Polyporales*68 |
|  | unclassified_*Polyporales*696 |
|  | *Volvariella* |
|  | *Blastobotrys* |
|  | unclassified_*Ascomycota*186 |
|  | unclassified_i3 |
| *Proteobacteria*; *Gammaproteobacteria*; *Legionellales*; *Coxiellaceae*; *Aquicella* | unclassified_*Peniophoracea*e |
| *Proteobacteria*; *Gammaproteobacteria*; *Xanthomonadales*; *Xanthomonadaceae*; *Dyella* | *Capronia* |
|  | *Cylindrobasidium* |
|  | *Nakazawaea* |

**BACTERIA EARLY Stage**

**Table S6.** AIC values for 6 rank abundance distribution models. Lowest AIC values for each sample represents the best fit model.

|  |  | **AIC^1^** | | | | | |
| --- | --- | --- | --- | --- | --- | --- | --- |
| **Sample** |  | **Broken-stick** | **Pre-Emption** | **Log-Normal** | **Zipf** | **Zipf-Mandelbrot** | **ZSM** |
| **Pi01.a** |  | 1506.6 | 319.06 | 455.06 | 518.39 | 241.59 | **231.95** |
| **Pi01.b** |  | 1573.2 | 299.28 | 428.76 | 477.72 | 237.78 | **190.06** |
| **Pi01.c** |  | 1462.7 | 299.26 | 537.26 | 621.34 | 303.26 | **185.24** |
| **Pi04.a** |  | 714.42 | 221.29 | 327.93 | 448.24 | 224.60 | **219.21** |
| **Pi04.b** |  | 785.44 | 246.91 | 318.21 | 421.24 | **209.89** | 220.79 |
| **Pi04.c** |  | 1101.2 | 339.13 | 443.02 | 541.23 | **262.95** | 306.42 |
| **Pi05.a** |  | 1047.6 | 542.33 | 368.72 | 419.84 | **280.26** | 459.56 |
| **Pi05.b** |  | 1000.4 | 493.41 | 33285 | 391.87 | **262.21** | 491.80 |
| **Pi05.c** |  | 1052.8 | 632.67 | 383.61 | 430.76 | **317.45** | 561.48 |

^1^AIC for radfit-generated models calculated the equation AIC = -2log-likehood + 2*npar. AIC was calculated in the same way for the zero-sum model, from the minimum of log-likehood reported by TeTame, then multiplied by -1 to obtain the maximum log-likehood value. * The best fit model with lowest AIC value.

**BACTERIA MID stage**

**Table S7.** AIC values for 6 rank abundance distribution models. Lowest AIC values for each sample represents the best fit model.

|  |  | **AIC^1^** | | | | | |
| --- | --- | --- | --- | --- | --- | --- | --- |
| **Sample** |  | **Broken-stick** | **Pre-Emption** | **Log-Normal** | **Zipf** | **Zipf-Mandelbrot** | **ZSM** |
| **Pi06.a** |  | 584.10 | 266.21 | 317.13 | 462.25 | **231.85** | 650.618 |
| **Pi06.b** |  | 718.09 | 344.44 | 323.31 | 434.62 | **245.18** | 727.858 |
| **Pi07.a** |  | 1168.4 | 450.13 | **228.79** | 271.35 | 240.44 | 515.154 |
| **Pi07.b** |  | 1078.6 | 425.24 | **191.52** | 233.50 | 215.02 | 467.764 |
| **Pi07.c** |  | 1193.0 | 492.79 | **237.94** | 278.23 | 251.12 | 557.714 |
| **Pi08.a** |  | 981.10 | 342.82 | 336.02 | 432.70 | **219.82** | 614.584 |
| **Pi08.b** |  | 880.36 | 307.91 | 318.27 | 414.27 | **202.86** | 539.014 |
| **Pi08.c** |  | 933.16 | 314.92 | 393.58 | 513.79 | **231.07** | 555.822 |
| **Pi09.a** |  | 623.29 | 315.22 | 272.96 | 377.34 | **239.18** | 584.452 |
| **Pi09.b** |  | 566.71 | 319.66 | **218.80** | 311.67 | 235.08 | 573.778 |
| **Pi09.c** |  | 519.88 | 286.84 | 250.96 | 357.27 | **230.24** | 542.63 |
| **Pi10.a** |  | 1303.4 | 562.63 | 212.81 | 207.85 | **186.57** | 537.914 |
| **Pi10.b** |  | 802.54 | 373.06 | 206.04 | 256.93 | **193.10** | 525.618 |
| **Pi10.c** |  | 1077.4 | 538.01 | **253.94** | 297.08 | 264.60 | 757.886 |
| **Pi11.a** |  | 454.51 | 237.00 | 298.94 | 462.83 | **235.73** | 648.766 |
| **Pi11.b** |  | 722.62 | 283.16 | 386.42 | 552.96 | **256.82** | 745.052 |
| **Pi11.c** |  | 440.22 | 232.37 | 274.37 | 422.41 | **221.70** | 624.294 |
| **Pi12.a** |  | 736.79 | 328.55 | 321.02 | 444.69 | **252.29** | 685.546 |
| **Pi12.b** |  | 779.34 | 329.36 | 336.22 | 463.67 | **252.53** | 710.432 |
| **Pi12.c** |  | 758.96 | 365.83 | 313.05 | 426.07 | **267.35** | 760.536 |

^1^AIC for radfit-generated models calculated the equation AIC = -2log-likehood + 2*npar. AIC was calculated in the same way for the zero-sum model, from the minimum of log-likehood reported by TeTame, then multiplied by -1 to obtain the maximum log-likehood value. * The best fit model with lowest AIC value.

**BACTERIA LATE stage**

**Table S8.** AIC values for 6 rank abundance distribution models. Lowest AIC values for each sample represents the best fit model.

|  |  | **AIC^1^** | | | | | |
| --- | --- | --- | --- | --- | --- | --- | --- |
| **Sample** |  | **Broken-stick** | **Pre-Emption** | **Log-Normal** | **Zipf** | **Zipf-Mandelbrot** | **ZSM** |
| **Pi13.a** |  | 661.80 | 560.31 | **346.78** | 399.44 | 357.79 | 888.766 |
| **Pi13.b** |  | 640.48 | 519.471 | **360.61** | 439.94 | 376.31 | 947.92 |
| **Pi13.c** |  | 631.03 | 551.93 | **353.77** | 419.19 | 391.77 | 883.086 |
| **Pi14.a** |  | 749.82 | 464.33 | **299.29** | 386.82 | 326.43 | 747.218 |
| **Pi14.b** |  | 850.79 | 552.30 | 348.36 | 416.84 | **340.83** | 912.856 |
| **Pi14.c** |  | 801.00 | 516.60 | **316.13** | 391.19 | 345.34 | 805.628 |
| **Pi15.a** |  | 731.48 | 619.51 | 436.00 | 492.96 | **415.32** | 1358.226 |
| **Pi15.b** |  | 643.28 | 598.88 | 474.61 | 550.24 | **466.09** | 1552.058 |
| **Pi15.c** |  | 611.09 | 589.64 | 434.84 | 481.69 | **405.74** | 1425.14 |
| **Pi16.a** |  | 764.73 | 402.79 | 273.99 | 351.22 | **241.80** | 671.226 |
| **Pi16.b** |  | 794.10 | 462.10 | 301.67 | 371.26 | **277.50** | 692.402 |
| **Pi16.c** |  | 772.10 | 428.08 | **289.47** | 379.23 | 290.46 | 704.454 |
| **Pi17.a** |  | 753.54 | 632.39 | 427.83 | 486.28 | **427.51** | 1129.948 |
| **Pi17.b** |  | 708.84 | 578.71 | 456.41 | 547.46 | **442.94** | 1138.132 |
| **Pi17.c** |  | 808.70 | 695.35 | 456.26 | 492.35 | **434.59** | 1151.822 |
| **Pi18.a** |  | 725.83 | 479.37 | 423.86 | 534.85 | **352.44** | 1024.142 |
| **Pi18.b** |  | 714.71 | 476.89 | 441.04 | 559.59 | **357.53** | 1064.942 |
| **Pi18.c** |  | 682.24 | 467.63 | 411.51 | 524.92 | **353.96** | 1018.226 |
| **Pi19.a** |  | 796.82 | 575.93 | 467.85 | 565.87 | **419.79** | 1135.962 |
| **Pi19.b** |  | 687.03 | 538.54 | 432.89 | 529.21 | **404.42** | 1077.644 |
| **Pi19.c** |  | 764.09 | 591.06 | 439.22 | 521.66 | **420.99** | 1102.834 |
| **Pi20.a** |  | 774.02 | 505.97 | 392.74 | 498.04 | **381.56** | 959.868 |
| **Pi20.b** |  | 704.00 | 463.53 | 369.54 | 476.88 | **352.29** | 930.98 |
| **Pi20.c** |  | 746.88 | 481.27 | 355.23 | 451.78 | **343.99** | 911.078 |

^1^AIC for radfit-generated models calculated the equation AIC = -2log-likehood + 2*npar. AIC was calculated in the same way for the zero-sum model, from the minimum of log-likehood reported by TeTame, then multiplied by -1 to obtain the maximum log-likehood value. * The best fit model with lowest AIC value.

**FUNGI EARLY stage**

**Table S9.** AIC values for 6 rank abundance distribution models. Lowest AIC values for each sample represents the best fit model.

|  |  | **AIC^1^** | | | | | |
| --- | --- | --- | --- | --- | --- | --- | --- |
| **Sample** |  | **Broken-stick** | **Pre-Emption** | **Log-Normal** | **Zipf** | **Zipf-Mandelbrot** | **ZSM** |
| **Pi01.a** |  | 3070.0 | 489.27 | 919.15 | 1036.3 | 493.27 | **365.61** |
| **Pi01.b** |  | 2579.5 | 733.76 | 1142.46 | 1376.5 | 737.76 | **246.34** |
| **Pi01.c** |  | 3082.1 | 401.98 | 829.69 | 941.86 | 405.98 | **353.80** |
| **Pi04.a** |  | 2578.7 | **273.31** | 776.73 | 1005.3 | 276.68 | 365.40 |
| **Pi04.b** |  | 2597.5 | 299.92 | 671.72 | 870.37 | **281.39** | 414.89 |
| **Pi04.c** |  | 2924.67 | 352.65 | 829.99 | 1028.1 | **331.49** | 465.80 |
| **Pi05.a** |  | 5969.8 | 474.83 | 162.73 | **102.93** | 104.93 | 425.45 |
| **Pi05.b** |  | 5440.4 | 337.16 | 146.29 | **99.338** | 101.33 | 357.08 |
| **Pi05.c** |  | 6300.8 | 587.38 | 189.30 | **119.12** | 121.12 | 482.60 |

^1^AIC for radfit-generated models calculated the equation AIC = -2log-likehood + 2*npar. AIC was calculated in the same way for the zero-sum model, from the minimum of log-likehood reported by TeTame, then multiplied by -1 to obtain the maximum log-likehood value. * The best fit model with lowest AIC value.

**FUNGI MIDDle stage**

**Table S10.** AIC values for 6 rank abundance distribution models. Lowest AIC values for each sample represents the best fit model.

|  |  | **AIC^1^** | | | | | |
| --- | --- | --- | --- | --- | --- | --- | --- |
| **Sample** |  | **Broken-stick** | **Pre-Emption** | **Log-Normal** | **Zipf** | **Zipf-Mandelbrot** | **ZSM** |
| **Pi06.a** |  | 4440.2 | 1225.2 | 325.18 | **310.52** | 312.52 | 746.13 |
| **Pi06.b** |  | 4664.3 | 1292.1 | 281.09 | **232.11** | 234.31 | 786.97 |
| **Pi06.c** |  | 4692.7 | 1052.5 | 229.05 | **199.73** | 201.13 | 870.68 |
| **Pi07.a** |  | 3346.8 | 701.28 | 436.75 | 564.74 | **265.61** | 968.92 |
| **Pi07.b** |  | 3145.0 | 849.47 | 388.28 | 492.09 | **276.34** | 985.03 |
| **Pi07.c** |  | 2996.7 | 635.05 | 390.99 | 503.35 | **257.88** | 773.73 |
| **Pi08.a** |  | 4513.9 | 1394.2 | 259.51 | **209.60** | 211.60 | 706.40 |
| **Pi08.b** |  | 4011.5 | 1138.9 | 248.41 | 222.38 | **199.84** | 716.29 |
| **Pi08.c** |  | 3729.1 | 1163.1 | 240.85 | **200.01** | 202.01 | 1032.2 |
| **Pi09.a** |  | 4293.9 | 1707.2 | 461.50 | **417.23** | 419.23 | 899.62 |
| **Pi09.b** |  | 5069.0 | 1651.6 | 364.36 | **306.52** | 308.52 | 646.18 |
| **Pi09.c** |  | 5202.3 | 1691.0 | 397.96 | **341.46** | 343.46 | 1076.3 |
| **Pi10.a** |  | 6475.6 | 1086.6 | 339.59 | **261.29** | 263.29 | 488.85 |
| **Pi10.b** |  | 6328.5 | 1193.9 | 381.70 | **303.27** | 305.27 | 470.16 |
| **Pi10.c** |  | 6140.7 | 1185.9 | 341.68 | **260.65** | 262.65 | 504.01 |
| **Pi11.a** |  | 6768.9 | 1819.4 | 392.92 | **273.96** | 275.96 | 955.11 |
| **Pi11.b** |  | 5795.5 | 1129.1 | 243.55 | **170.81** | 172.81 | 683.59 |
| **Pi11.c** |  | 5504.6 | 869.70 | 204.35 | **145.50** | 147.50 | 542.73 |
| **Pi12.a** |  | 6541.3 | *nd* | 296.16 | **217.27** | 219.27 | 363.30 |
| **Pi12.b** |  | 7156.3 | *nd* | 351.80 | **259.29** | 261.29 | 501.32 |
| **Pi12.c** |  | 7198.0 | *nd* | 376.83 | **281.59** | 283.59 | 448.41 |

^1^AIC for radfit-generated models calculated the equation AIC = -2log-likehood + 2*npar. AIC was calculated in the same way for the zero-sum model, from the minimum of log-likehood reported by TeTame, then multiplied by -1 to obtain the maximum log-likehood value. * The best fit model with lowest AIC value. *nd* not determined

**FUNGI LATE stage**

**Table S11.** AIC values for 6 rank abundance distribution models. Lowest AIC values for each sample represents the best fit model.

|  |  | **AIC^1^** | | | | | |
| --- | --- | --- | --- | --- | --- | --- | --- |
| **Sample** |  | **Broken-stick** | **Pre-Emption** | **Log-Normal** | **Zipf** | **Zipf-Mandelbrot** | **ZSM** |
| **Pi13.a** |  | 4396.8 | 1258.9 | 920.83 | 919.76 | **592.15** | 835.55 |
| **Pi13.b** |  | 4662.1 | 1189.0 | 629.55 | 583.30 | **427.61** | 715.35 |
| **Pi13.c** |  | 4641.5 | 1597.1 | 582.89 | 483.91 | **446.69** | 693.99 |
| **Pi14.a** |  | 7939.1 | 3048.3 | 794.43 | **601.00** | 603.00 | 622.09 |
| **Pi14.b** |  | 8874.8 | 3493.3 | 856.06 | **627.35** | 629.35 | 757.82 |
| **Pi14.c** |  | 7978.8 | 3259.4 | 880.87 | **670.84** | 672.84 | 675.84 |
| **Pi15.a** |  | 5772.9 | *nd* | 177.47 | **125.02** | 127.02 | 238.21 |
| **Pi15.b** |  | 4466.5 | *nd* | 99.917 | **66.271** | 68.271 | 175.86 |
| **Pi15.c** |  | 4054.1 | *nd* | 83.815 | **57.085** | 59.085 | 133.52 |
| **Pi16.a** |  | 3513.8 | 1907.7 | 596.57 | **638.04** | 640.04 | 1284.7 |
| **Pi16.b** |  | 3412.2 | 1655.3 | 601.16 | **694.01** | 696.01 | 1008.7 |
| **Pi16.c** |  | 3100.2 | 1608.4 | 567.65 | **641.80** | 643.80 | 862.04 |
| **Pi17.a** |  | 6070.0 | 1939.1 | 381.05 | **229.86** | 231.86 | 611.50 |
| **Pi17.b** |  | 5034.5 | 1116.2 | 712.80 | 636.26 | **540.68** | 745.66 |
| **Pi17.c** |  | 5954.3 | 1923.6 | 366.66 | **236.86** | 238.86 | 594.40 |
| **Pi18.a** |  | 3644.7 | 991.93 | 1051.7 | 1239.1 | **529.20** | 1211.4 |
| **Pi18.b** |  | 3302.2 | 1016.3 | 776.12 | 921.08 | **414.96** | 1276.0 |
| **Pi18.c** |  | 2788.5 | 611.15 | 874.44 | 1153.6 | **378.19** | 1106.6 |
| **Pi19.a** |  | 4122.8 | 875.24 | 1183.3 | 1261.6 | 684.76 | **516.48** |
| **Pi19.b** |  | 4392.4 | 967.45 | 1124.0 | 1179.0 | 702.45 | **554.80** |
| **Pi19.c** |  | 4321.7 | 974.45 | 1442.2 | 1548.3 | 858.84 | **508.63** |
| **Pi20.a** |  | 5180.9 | 1528.9 | 1321.9 | 1299.5 | 859.19 | **829.85** |
| **Pi20.b** |  | 5026.6 | 1514.0 | 974.48 | 914.87 | **631.25** | 921.04 |
| **Pi20.c** |  | 3995.8 | 1056.8 | 461.50 | 435.48 | **316.84** | 655.68 |

^1^AIC for radfit-generated models calculated the equation AIC = -2log-likehood + 2*npar. AIC was calculated in the same way for the zero-sum model, from the minimum of log-likehood reported by TeTame, then multiplied by -1 to obtain the maximum log-likehood value. * The best fit model with lowest AIC value.

**
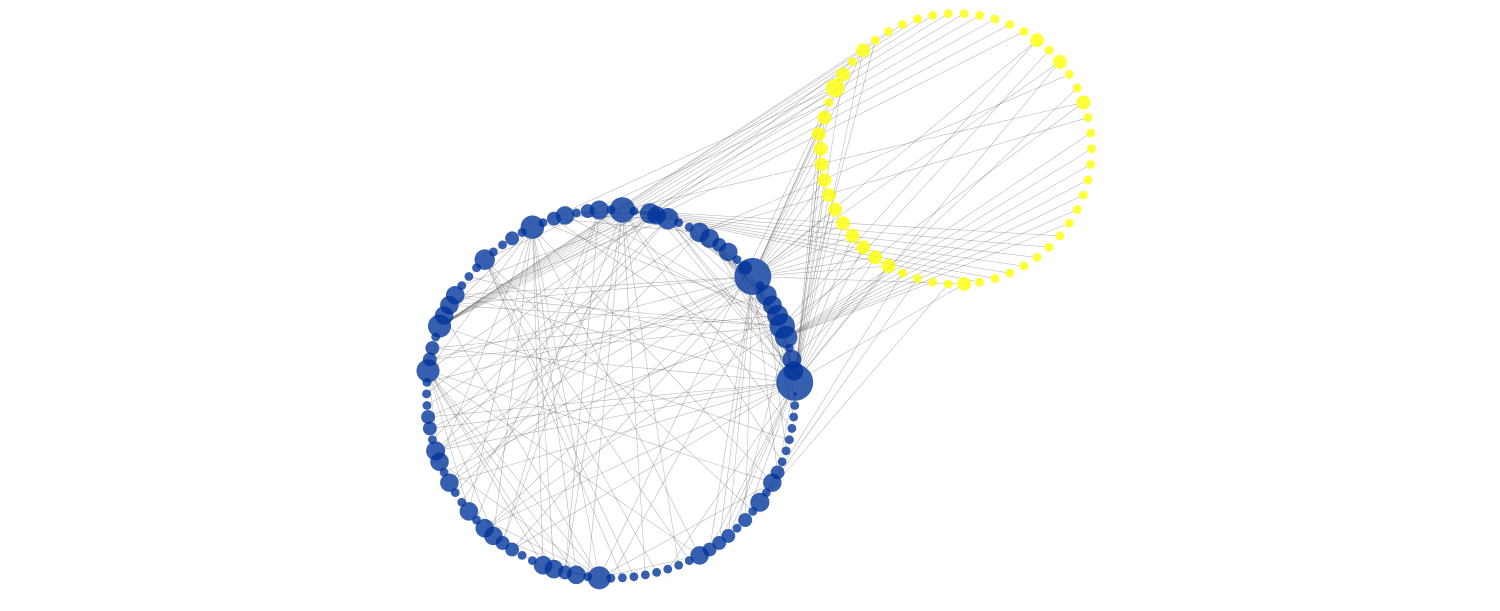
**

**Figure S1.** Network co-occurrence analysis of bacterial and fungal genera within decaying wood samples. Connections represent Pearson’s correlations (p > 0.8 and P-value ≤ 0.05). Blue nodes represent bacterial genera and yellow fungal genera. The size of nodes is proportional to degree centrality.
